# Supplementary material for: Can Donkey Behavior and Cognition Be Used to Trace Back, Explain, or Forecast Moon Cycle and Weather Events?
Source: Animals (Basel). 2018 Nov 19;8(11):215. doi: 10.3390/ani8110215 (PMC6262452; doi:10.3390/ani8110215)
Supplement: Supplementary file 1 [file animals-08-00215-s001.zip › Supplementary Table S1.docx]

**Can donkey behavior and cognition be used to trace back, explain or forecast moon cycle and weather events?**

Francisco Javier Navas González, Jordi Jordana Vidal, Gabriela Pizarro Inostroza, Ander Arando Arbulu, Juan Vicente Delgado Bermejo

Animals

*Department of Genetics, Faculty of Veterinary Sciences, University of Córdoba, Córdoba.*

[fjng87@hotmail.com](mailto:fjng87@hotmail.com)

**Supplementary Table S1.** Category description and definition for response type, intensity of response, mood/emotion, and learning variables directly controlled during the operant conditioning test.

| Behavioral categorical variables | Definition | Categories/Scale |
| --- | --- | --- |
| Type of response | We classified the animals according to the coping strategy that they implemented. This is whether the donkeys did not pay attention to the stimulus presented or they adopted a reactive or proactive strategy towards it. | Hyporeactive, neutral, hyperreactive. |
| Mood/emotion | By mood/emotion we refer to the emotional/psychological state of the donkey. This emotional/psychological state can last for a short or a longer period of time and is usually a result of an external stimulus as those presented at our test. | Distracted, dejected/depressed, indifferent/unresponsive, calm, awaiting, curious, cautious, mistrustful, surprised, nervous, fearful, rejective. |
| Intensity of response | The response intensity scale measured the degree at which the emotional/psychological states in the first scale mentioned were displayed. | Low, mid-low, mid, mid-high, high. |
| Learning | We assessed donkeys’ extinction/learning processes, studying the success rate of the donkeys at completing the operant conditioning test to which they were exposed. We studied extinction learning processes [55], rather than habituation learning processes [56, 57] as for the second forms of learning, the donkeys may decrease or cease their responses to each stimulus after repeated or prolonged presentations, not because of the reinforcement event. | Stops and refuses to cross, dodges the surface, erratically crosses laterally deviating if compelled to do it, crosses but shows doubt signs, crosses completely without problems. |
| For type of response, mood/emotion and intensity of response we followed the definitions and scales provided in Navas et al. [9]. | | |
