# Supplementary material for: Can Donkey Behavior and Cognition Be Used to Trace Back, Explain, or Forecast Moon Cycle and Weather Events?
Source: Animals (Basel). 2018 Nov 19;8(11):215. doi: 10.3390/ani8110215 (PMC6262452; doi:10.3390/ani8110215)
Supplement: Supplementary file 1 [file animals-08-00215-s001.zip › Supplementary Table S2.docx]

**Can donkey behavior and cognition be used to trace back, explain or forecast moon cycle and weather events?**

Francisco Javier Navas González, Jordi Jordana Vidal, Gabriela Pizarro Inostroza, Ander Arando Arbulu, Juan Vicente Delgado Bermejo

Animals

*Department of Genetics, Faculty of Veterinary Sciences, University of Córdoba, Córdoba.*

[fjng87@hotmail.com](mailto:fjng87@hotmail.com)

**Supplementary Table S2.** Categorical variable description and levels for the effects of meteorological environment and birth characteristics collaterally controlled during the fulfilment of the test during the first phase of the study.

| Cluster | Variable | Level |
| --- | --- | --- |
| Meteorological and environmental conditions | Year of evaluation | 2013, 2014, 2015 |
|  | Season of evaluation | Cold season, hot season |
|  | Weather conditions | Sunny, Cloudy |
|  | Temperature | 12º to 18ºC (Cool), 19º to 24ºC (Mild), 25 to 29 ºC (Hot) |
|  | Moon phase at evaluation | New moon, waxing crescent, waxing gibbous, first quarter, last quarter, waning gibbous, waning crescent, full moon [[54](#_ENREF_54)] |
|  | Relative humidity | <50% (Low), 50% (Optimal), 51-55% (Medium), >55% (High) [[5](#_ENREF_5)] |
|  | Windspeed | 0 m/s (No wind), ≤3 m/s (Light speed wind), 5 to 8 m/s (Medium speed wind), ≥9 m/s (High speed wind) |
|  | Sunlight hours | <12 h (Less than half a day), 12 (Half a day), <12 (More than half a day) |
|  | Barometric pressure | <1013 hPa (Low), 1013-1015 hPa (Normal/Medium), >1015 hPa (High) |
|  | Rainfall on the day of evaluation | 0 mm (Absence), 0.1 to 2.8 mm (Slight), >2.9 mm (Moderate) |
|  | Rainfall on the following day | 0 mm (Absence), 0.1 to 2.8 mm (Almost absent), 2.9 to 10 mm (Slight), >10 mm (Moderate) |
| Animal birth characteristics | Season of birth | Winter, Spring, Summer, Autumn |
|  | Year of birth | 1990, 1992, 1994, 1996-2012 |
|  | Moon phase at birth | New moon, waxing crescent, waxing gibbous, first quarter, last quarter, waning gibbous, waning crescent, full moon [[54](#_ENREF_54)] |
