# Supplementary material for: Can Donkey Behavior and Cognition Be Used to Trace Back, Explain, or Forecast Moon Cycle and Weather Events?
Source: Animals (Basel). 2018 Nov 19;8(11):215. doi: 10.3390/ani8110215 (PMC6262452; doi:10.3390/ani8110215)
Supplement: Supplementary file 1 [file animals-08-00215-s001.zip › Supplementary Table S4.docx]

| **Can donkey behavior and cognition be used to trace back, explain or forecast moon cycle and weather events?** |
| --- |
| Francisco Javier Navas González, Jordi Jordana Vidal, Gabriela Pizarro Inostroza, Ander Arando Arbulu, Juan Vicente Delgado Bermejo |
| Animals |
| *Department of Genetics, Faculty of Veterinary Sciences, University of Córdoba, Córdoba.* |
| [fjng87@hotmail.com](mailto:fjng87@hotmail.com) |

**Supplementary Table S4.** Description for the mood and response type behavioral categorical variables and “Mercalli” scales.

| Mercalli scale | Mood/Attitude | Mercalli scale | Response type | Attitude towards the element presented |
| --- | --- | --- | --- | --- |
| 1 | Distracted | 1 | Hyporeactive | Pays attention^a^ and moves towards other elements around, without paying attention to the elements^c^ presented in the test. |
| 2 | Dejected/Depressed^b^ | 1 | Hyporeactive | Overall, body posture shows lowered head and neck, roundness to spine and tucked tail. It does not pay attention to any elements around. |
| 3 | Indifferent/Nonresponsive | 1 | Hyporeactive | Normal posture. Pays no attention to the element presented, but it is not distracted by other elements around. |
| 4 | Calm | 2 | Neutral | Does not get startled. Stands still. Pays attention to other elements around at the same time that it pays attention to the element presented. |
| 5 | Awaiting | 2 | Neutral | Does not get startled. Stands still. Only focuses on the element presented. |
| 6 | Curious | 2 | Neutral | Does not get startled. Stands still. Only focuses on the element presented. Moves its head towards the element presented. |
| 7 | Cautious | 2 | Neutral | Does not get startled. Pays attention and moves slightly towards the element (less than 1 m). |
| 8 | Mistrustful | 2 | Neutral | Does not get startled. Pays attention to and moves towards the element until approaching it completely. |
| 9 | Surprised | 3 | Hyperreactive | Only focused on the element being presented.  Gets startled but moves towards the element. |
| 10 | Nervous | 3 | Hyperreactive | Only focused on the element being presented.  Gets startled and tries to move away from the element presented at first. Able to move towards the element presented if led by the operator. |
| 11 | Fearful | 3 | Hyperreactive | Gets startled. Only focused on the element being presented.  Tries to move away from the element presented. Unable to move towards the element presented if led by the operator. |
| 12 | Rejection | 3 | Hyperreactive | Only focused on the element being presented.  Gets startled and moves away from the element presented noticeably. Pulls away from the leading rope when the operator tries to move towards the element presented. |
| ^a^By paying attention we mean that the donkey held direct visual contact with and/or directed its ear/s towards the element being presented.  ^b^All the animals displaying a dejected/depressed status had been born after the last third of their gestation had taken place during the cold wave occurring in Spain in 2005. Studies in rats have reported that the pregnancies of mothers who had been exposed to extreme cold conditions presented a resulting offspring at increased risk to experience future developmental, learning and emotional disorders.  ^c^Elements presented in the test are described in Table 2.  Accessed from [Navas et al. 2018](#_ENREF_44). | | | | |
